# Supplementary material for: Structure and mechanism of NALCN-FAM155A-UNC79-UNC80 channel complex
Source: Nat Commun. 2022 May 12;13:2639. doi: 10.1038/s41467-022-30403-7 (PMC9098444; doi:10.1038/s41467-022-30403-7)
Supplement: Supplementary file 1 — Supplementary Information [file 41467_2022_30403_MOESM1_ESM.pdf]

Supplementary Information for

Structure and mechanism of NALCN-FAM155A-UNC79-UNC80 channel complex

This PDF file contains:

Supplementary Figs. 1-8

Supplementary Table 1

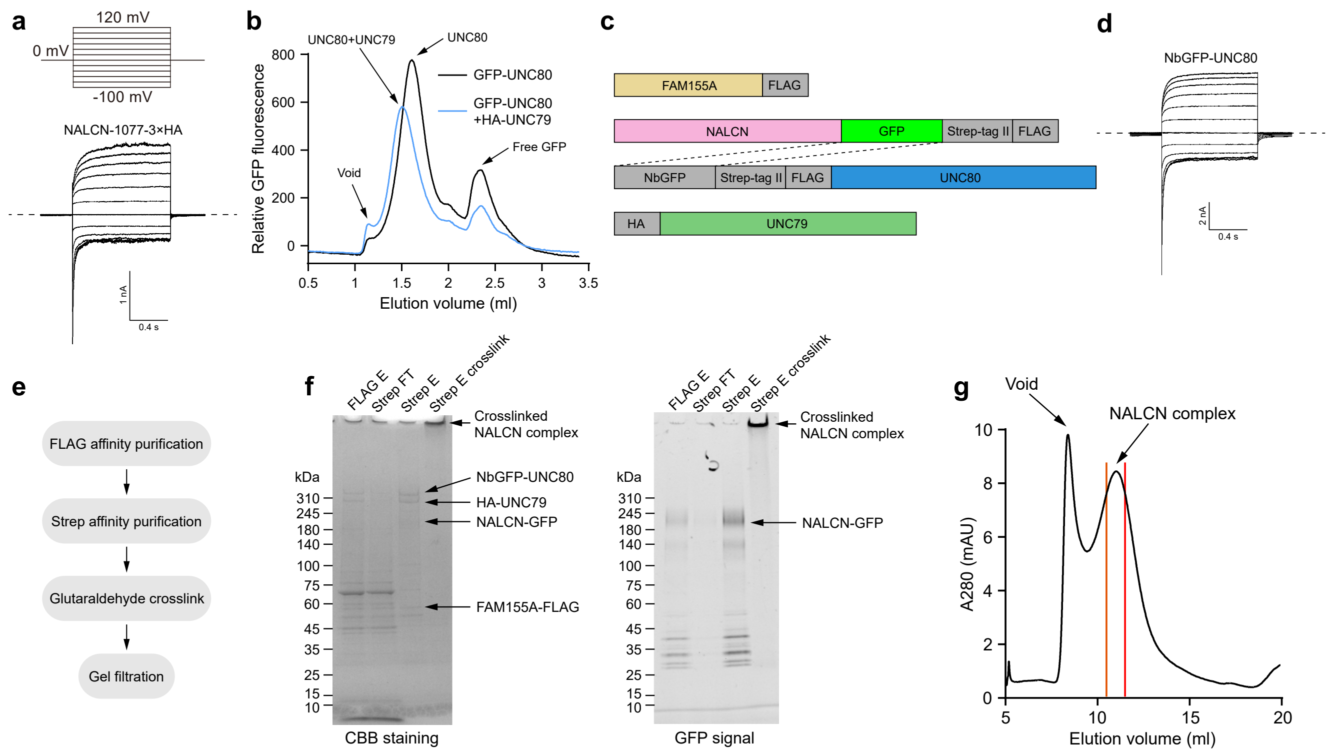

Supplementary Fig. 1 Electrophysiology and biochemistry characterization of NALCN-FAM155A-UNC79-UNC80 quaternary complex. a The whole-cell voltage step protocol and representative whole-cell currents of NALCN with 3×HA tag inserted between residues 1077 and 1078 in the presence of FAM155A, UNC79, and UNC80. Dashed lines indicate the position of 0 nA. The experiments were repeated independently three times with similar results. **b** Fluorescence-detection size-exclusion chromatography of GFP-UNC80 in the absence (black) or presence of UNC79 (blue). The experiments were repeated independently three times with similar results. **c** The construct used for protein purification. **d** Representative whole-cell currents of constructs shown in **c**. The whole-cell voltage step protocol was same as in **a**. Dashed lines indicate the position of 0 nA. The experiments were repeated independently three times with similar results. **e** The protein purification work-flow. **f** SDS-PAGE of protein purified by affinity-chromatography stained by coomassie blue (left) or detected by in-gel fluorescence using GFP channel (right). The experiments were repeated independently twice with similar results. **g** Size-exclusion chromatography of crosslinked NALCN complex. Fractions between red lines were pooled for cryo-EM sample preparation. The experiments were repeated independently twice with similar results.

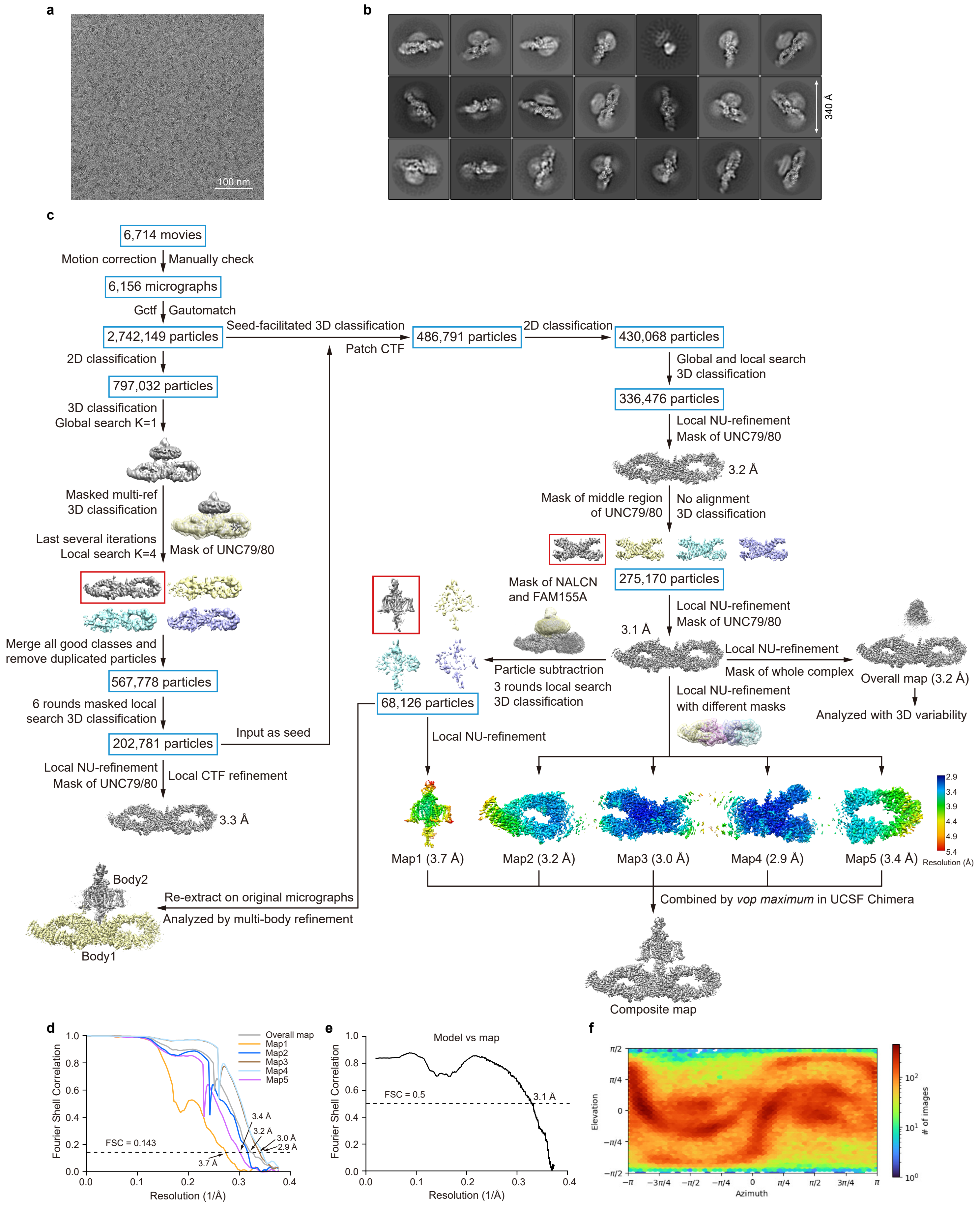

Supplementary Fig. 2 Cryo-EM image analysis of NALCN-FAM155A-UNC79-UNC80 quaternary complex. **a** Representative raw micrograph (6,714 in total) of NALCN-FAM155A-UNC79-UNC80 quaternary complex. **b** 2D-class averages of NALCN-FAM155A-UNC79-UNC80 quaternary complex with clear features. **c** Cryo-EM data processing workflow. For details, see 'Cryo-EM image analysis' in the Methods section. **d** Gold-standard Fourier Shell Correlation (FSC) of focus-refined maps shown in c after correction of masking effects. **e** FSC curve of the refined model versus EM map (low-pass filtered to 2.8 Å). **f** Angular distribution of consensus refinement of the quaternary complex.

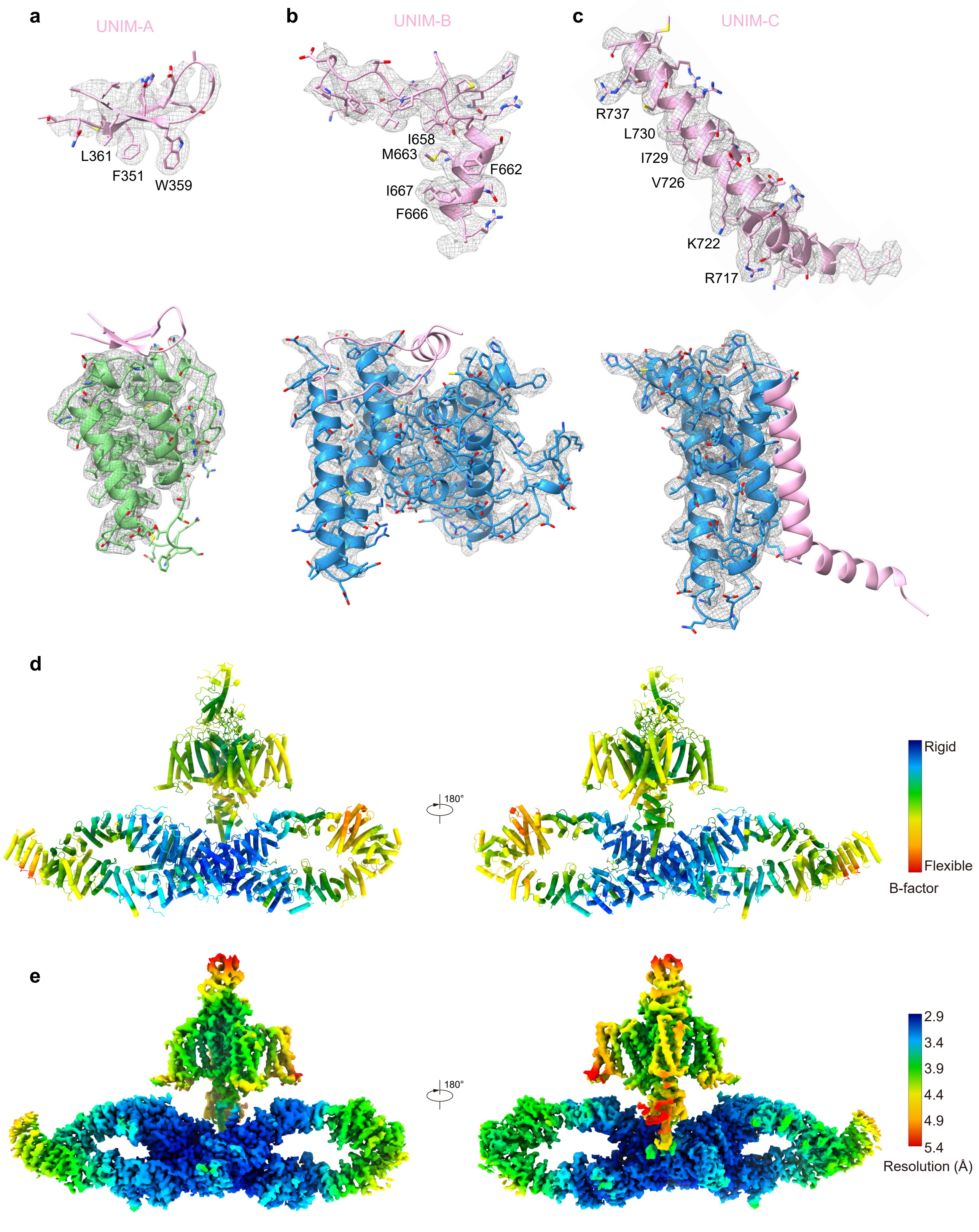

Supplementary Fig. 3 Electron density maps. a The electron density map of UNIM-A was shown in gray meshes, with the electron density of its interacting region in UNC79 is shown below. **b** The electron density map of UNIM-B was shown in gray mesh, with the electron density of its interacting region in UNC80 is shown below. **c** The electron density map of UNIM-C was shown in gray mesh, with the electron density of its interacting region in UNC80 is shown below. The contour level was 4-6 σ. **d** B-factor representation of NALCN-FAM155A-UNC79-UNC80 quaternary complex. **e** Composite local resolution map of NALCN-FAM155A-UNC79-UNC80 quaternary complex. The local resolution maps of focus refinement were combined by *vop minimum* in UCSF Chimera to generate composite local resolution map.

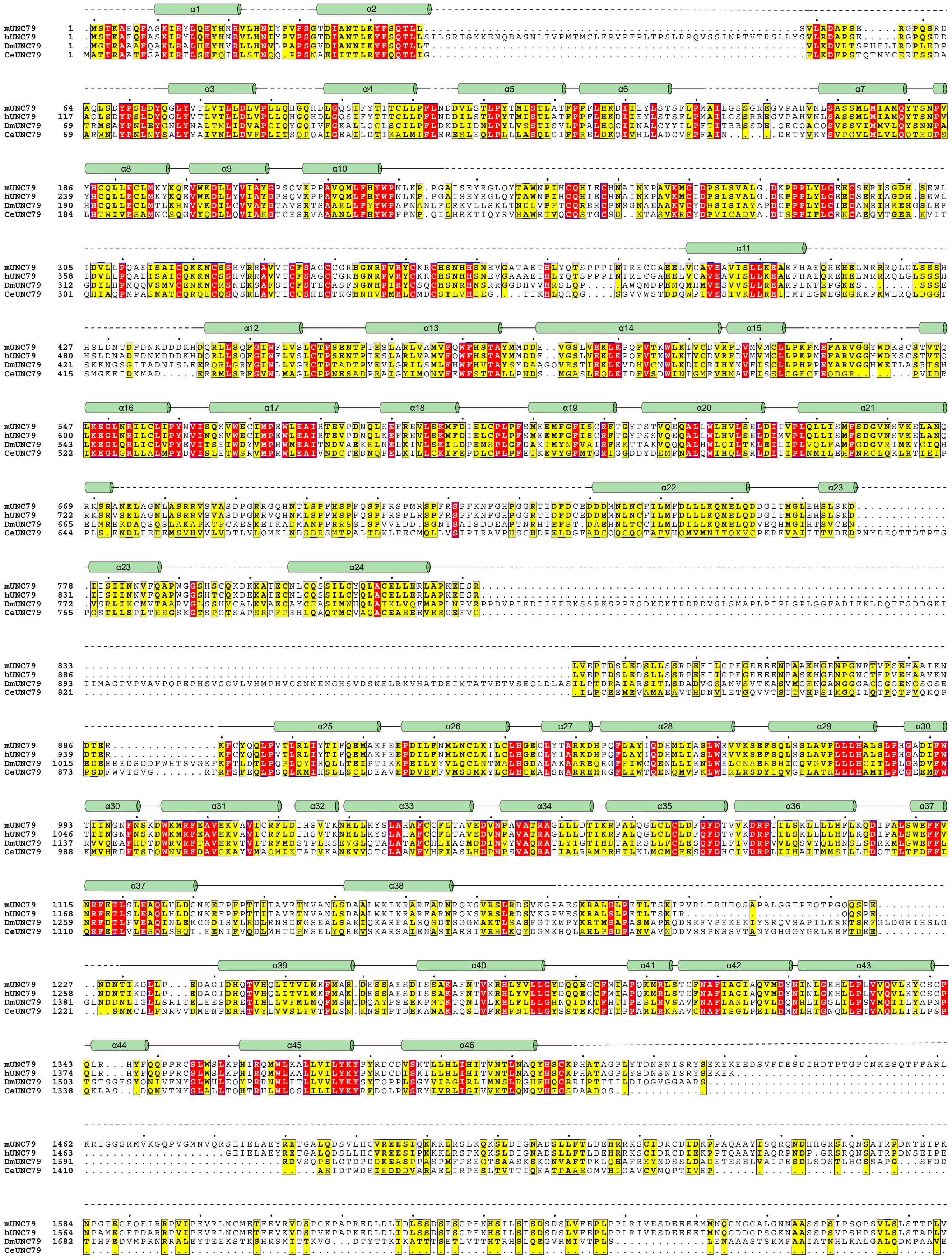
mUNC79
hUNC79
DmUNC79
CeUNC79
α1 α2 α3 α4 α5 α6 α7 α8 α9 α10 α11 α12 α13 α14 α15 α16 α17 α18 α19 α20 α21 α22 α23 α24 α25 α26 α27 α28 α29 α30 α31 α32 α33 α34 α35 α36 α37 α38 α39 α40 α41 α42 α43 α44 α45 α46

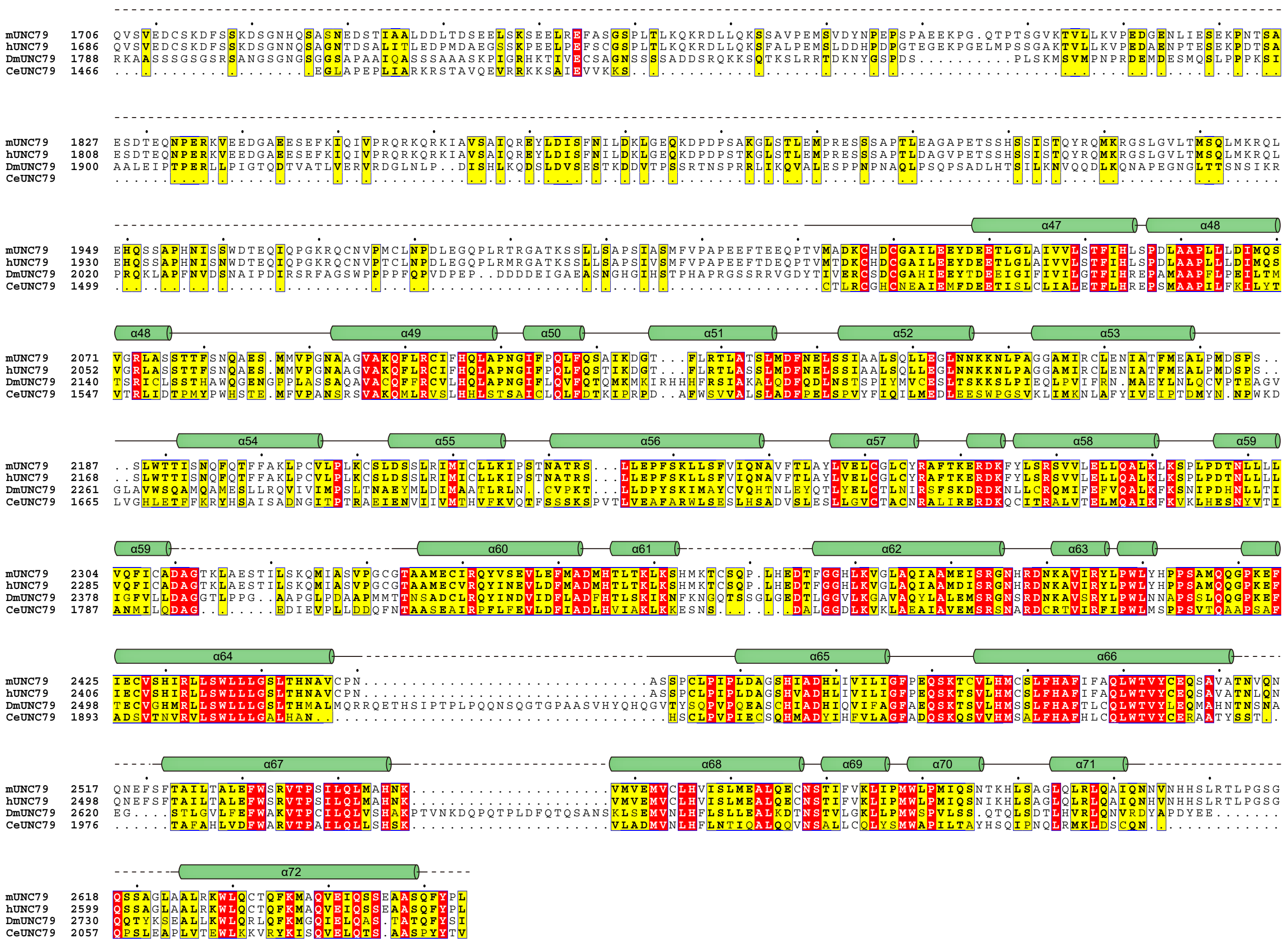

Supplementary Fig. 4 Sequence alignment of UNC79. The sequence alignment of UNC79 from mouse (mUNC79), human (hUNC79), Drosophila melanogaster (DmUNC79), and Caenorhabditis elegans (CeUNC79). Highly conserved and relative conserved residues are shaded in red and yellow, respectively. Secondary structures are shown above and unresolved residues are shown as dashes.

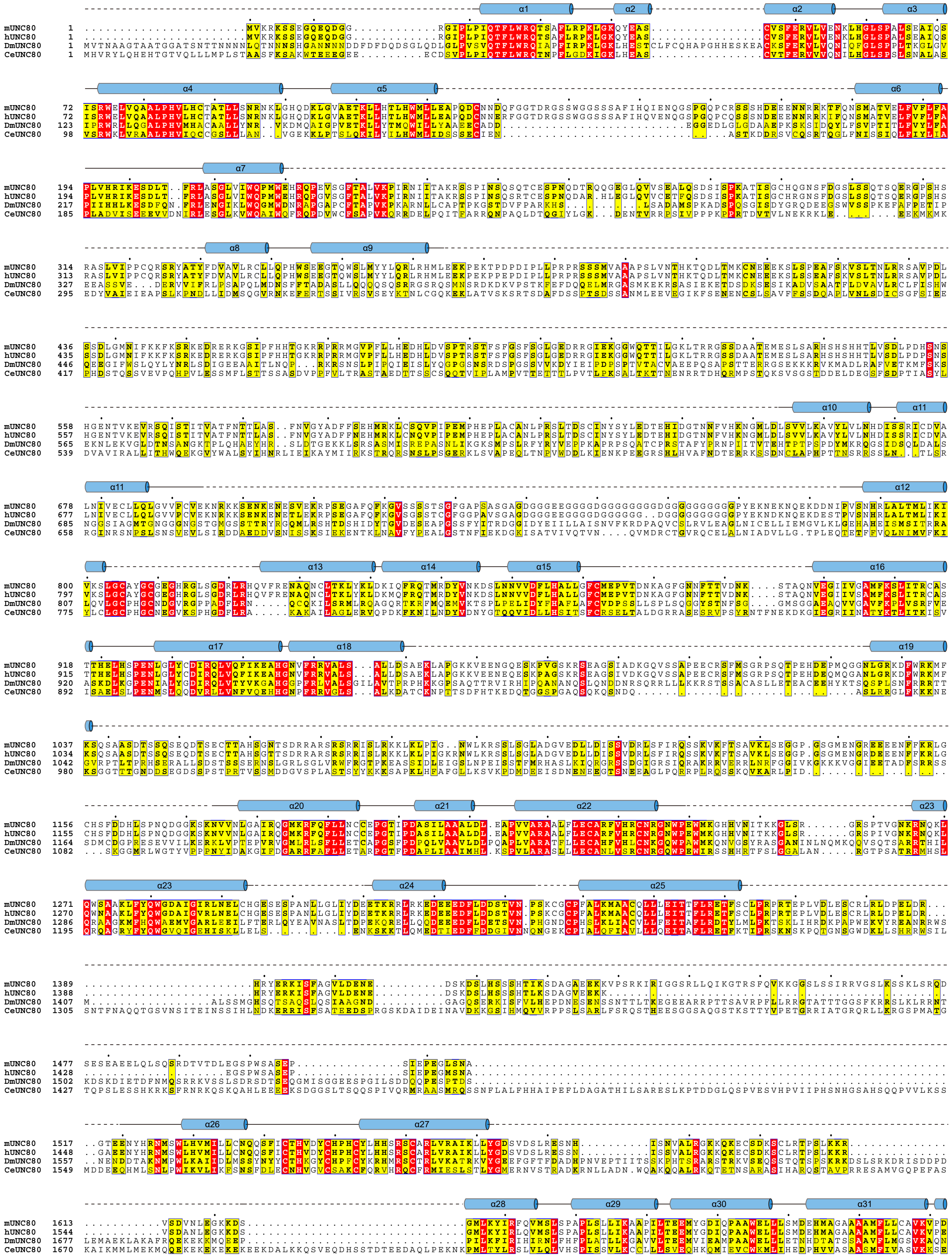
mUNC80
hUNC80
DmUNC80
CeUNC80
α1
α2
α3
α4
α5
α6
α7
α8
α9
α10
α11
α12
α13
α14
α15
α16
α17
α18
α19
α20
α21
α22
α23
α24
α25
α26
α27
α28
α29
α30
α31

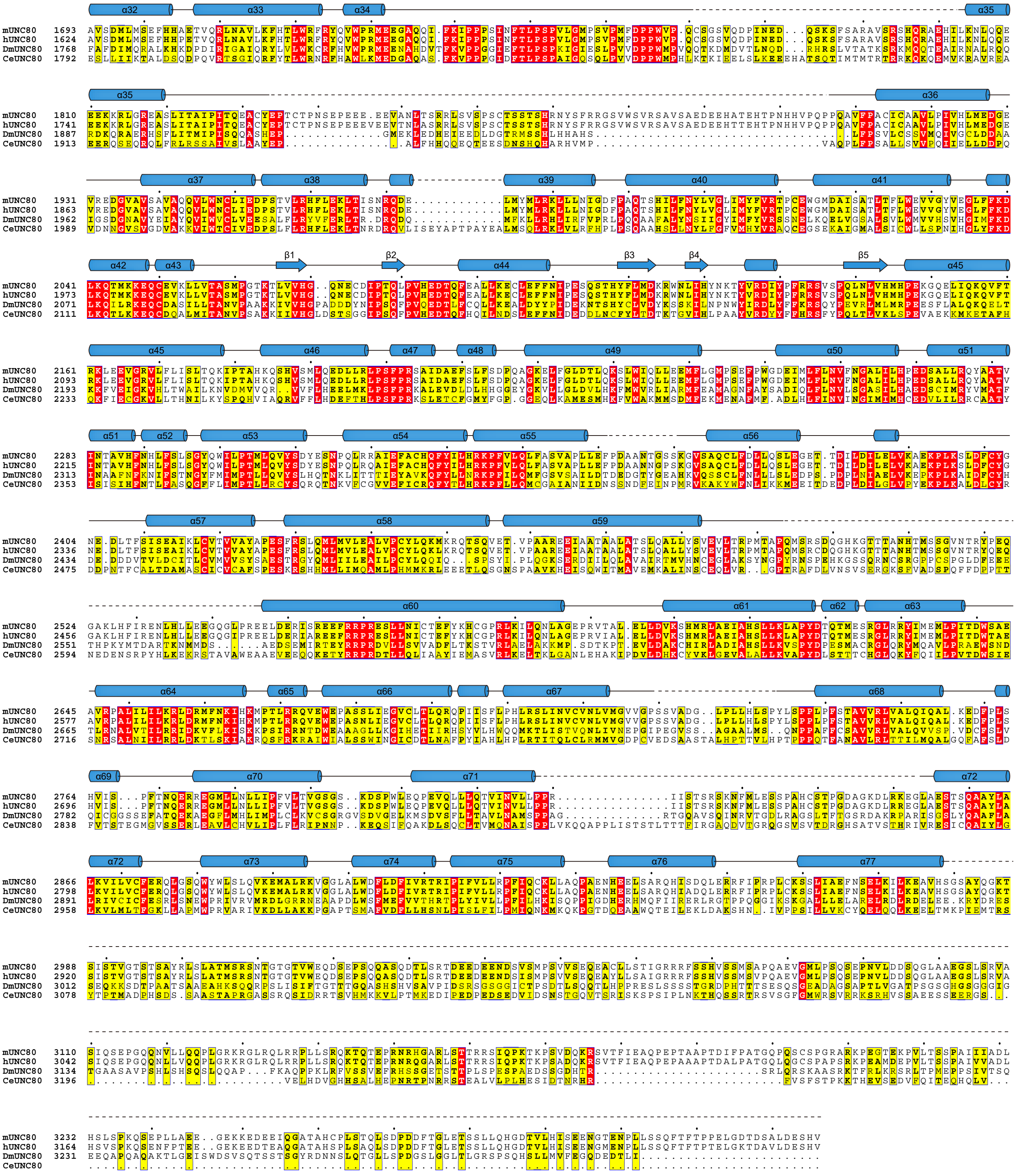

Supplementary Fig. 5 Sequence alignment of UNC80. The sequence alignment of UNC80 from mouse (mUNC80), human (hUNC80), Drosophila melanogaster (DmUNC80), and Caenorhabditis elegans (CeUNC80). Highly conserved and relative conserved residues are shaded in red and yellow, respectively. Secondary structures are shown above and unresolved residues are shown as dashes.

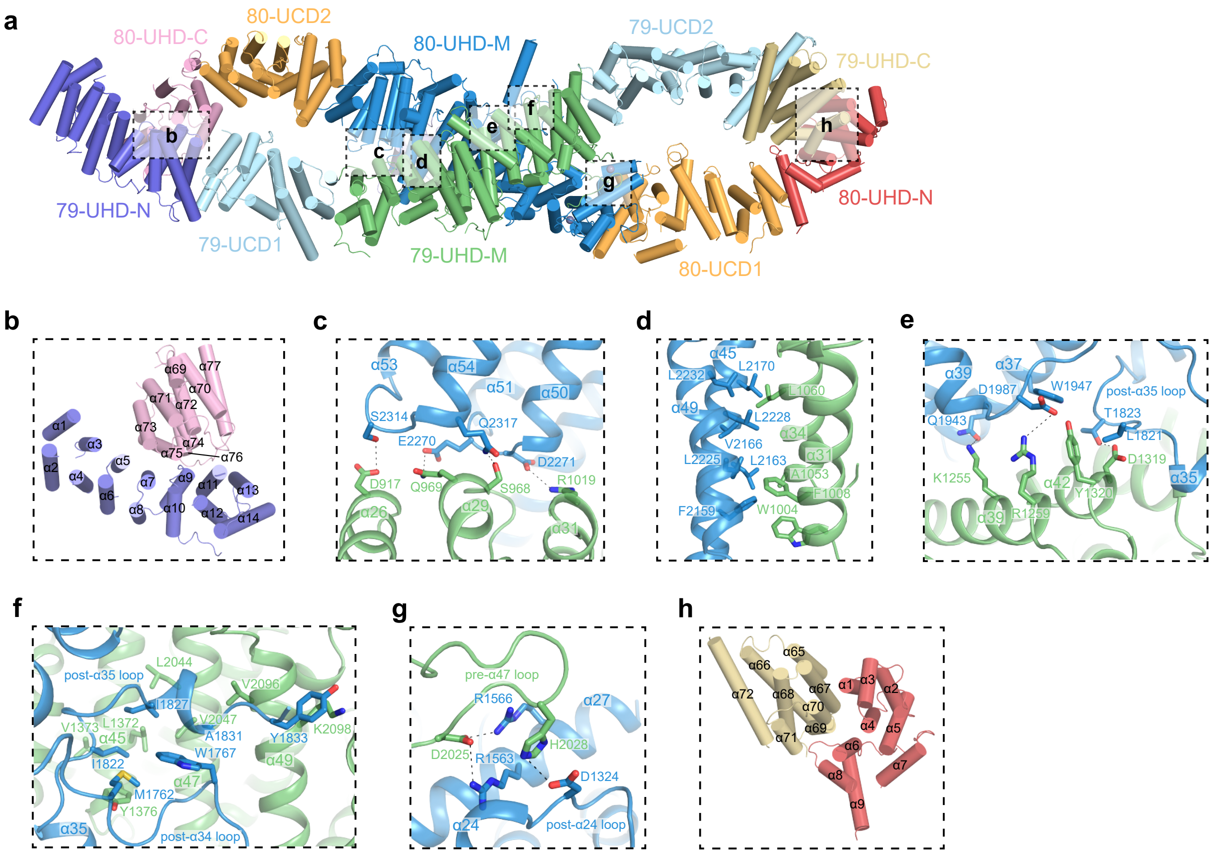

Supplementary Fig. 6 Details of interactions between UNC79 and UNC80. a Cartoon representation of UNC79-UNC80 heterodimer. Helices are shown as cylinders. Each domain is colored as in Fig. 2a. **b** The interface between 79-UHD-N and 80-UHD-C boxed in **a**. **c-g** The interface between 79-UHD-M and 80-UHD-M boxed in **a**. **h** The interface between 79-UHD-C and 80-UHD-N boxed in **a**.

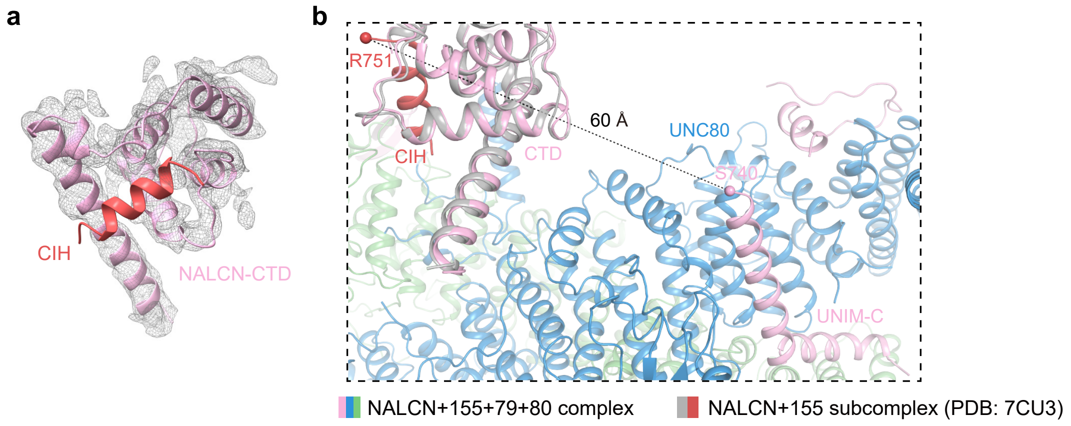

Supplementary Fig. 7 The Structure of NALCN CTD in NALCN-FAM155A-UNC79-UNC80 quaternary complex. a Electron density map of NALCN CTD was shown in gray meshes. The density of CIH is absent. The position of CIH (red helix) is based on the structure NALCN-FAM155A subcomplex (PDB ID: 7CU3). The contour level was 4 σ. **b** The structure of the NALCN-FAM155A subcomplex (PDB ID: 7CU3, gray and red) is aligned onto the NALCN-FAM155A-UNC79-UNC80 quaternary complex using the NALCN subunit as reference. The distance between Cα atoms of the N-terminus of CIH (R751) in the NALCN-FAM155A subcomplex and the C-terminus of UNIM-C (S740) is measured and labelled as dashes.

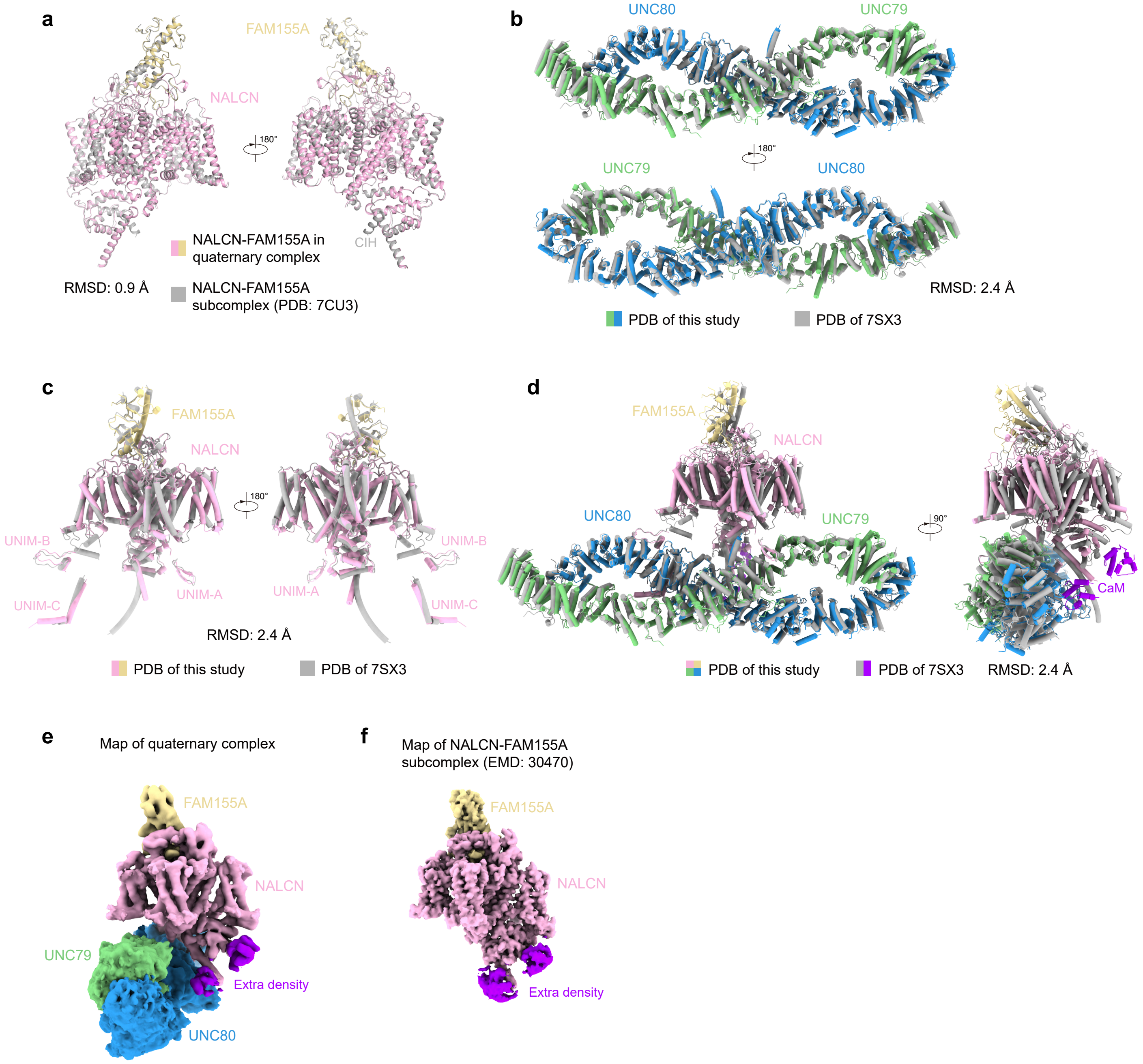

Supplementary Fig. 8 Structural comparison. **a** Structural comparison of NALCN-FAM155A subunits in NALCN-FAM155A-UNC79-UNC80 quaternary complex and NALCN-FAM155A subcomplex (PDB: 7CU3). For clarity, intracellular UNIMs were omitted. **b** Structural comparison of UNC79-UNC80 heterodimer between this study and 7SX3. **c** Structural comparison of NALCN-FAM155A subunits between this study and 7SX3. **d** Overall structural comparison of NALCN channel complex between this study and 7SX3. **e** Unsharpened cryo-EM map of NALCN-FAM155A-UNC79-UNC80 quaternary complex. Extra density around CTD of NALCN was highlighted with purple. The contour level was 4 σ. **f** Unsharpened cryo-EM map of NALCN-FAM155A subcomplex (EMD: 30470). Extra density around CTD of NALCN was highlighted with purple. The contour level was 4 σ.

Supplementary Table 1 Cryo-EM data collection, refinement and validation statistics.

	NALCN-FAM155A-UNC79-UNC80 complex
PDB ID	7W7G
EMDB ID	EMD-32344
**Data collection and processing**	
Magnification	105,000 ×
Voltage (kV)	300
Electron exposure (e⁻/Å^2)	50
Defocus range (μm)	-1.5 to -1.8
Pixel size (Å)	1.045
Symmetry imposed	*C1*
Initial particle images (no.)	2,742,149
Final particle images (no.)	68,126/275,170[#]
Map resolution (Å)	3.7/3.2/3.0/2.9/3.4[*]
FSC threshold	0.143
Map resolution range (Å)	250.0-2.9
**Refinement**	
Initial model used	7CU3 and AlphaFold2 prediction
Model resolution (Å)	3.1
FSC threshold	0.5
Model resolution range (Å)	250.0-3.1
Map sharpening *B* factor (Å^2)	-136.1/-103.1/-111.7/-110.3/-115.5[*]
Model composition	
Non-hydrogen atoms	38,143
Protein	4,758
Ligand	5
B factors (Å^2)	
Protein	96.29
Ligand	136.02
R.m.s. deviations	
Bond lengths (Å)	0.004
Bond angles (°)	0.516
Validation	
MolProbity score	2.09
Clashscore	8.02
Poor rotamers (%)	3.29
Ramachandran plot	
Favored (%)	96.12
Allowed (%)	3.88
Disallowed (%)	0.00

#The numbers of final particle images correspond to map1/map2-5 in Supplementary Fig. 2c.
*The numbers of map resolution and map sharpening *B* factor correspond to map1/2/3/4/5 in Supplementary Fig. 2c.
