# Supplementary material for: Structure and mechanism of NALCN-FAM155A-UNC79-UNC80 channel complex
Source: Nat Commun. 2022 May 12;13:2639. doi: 10.1038/s41467-022-30403-7 (PMC9098444; doi:10.1038/s41467-022-30403-7)
Supplement: Supplementary file 2 — Description of Additional Supplementary Files [file 41467_2022_30403_MOESM2_ESM.pdf]

File name: **Supplementary Movie 1**

Description: **The relative motion between NALCN-FAM155A subcomplex and UNC79-UNC80 heterodimer revealed by multi-body analysis.** Repositioning of reconstructed densities of NALCN FAM155A and UNC79-UNC80 along the first and the second eigenvectors reveals the relative motions between NALCNFAM155A and UNC79-UNC80. Eigenvector #1 explains 29% variance and eigenvector #2 explains 25% variance.

File name: **Supplementary Movie 2**

Description: **The motions within UNC79-UNC80 heterodimer revealed by 3D variability.** 3D variability analysis after consensus refinement in cryoSPARC.

File name: **Supplementary Movie 3**

Description: **Overall structure of NALCN-FAM155AUNC79-UNC80 quaternary complex.** The cryo-EM map of NALCNFAM155A-UNC79-UNC80 quaternary complex.
